# Antifungal Use in Perforated Peptic Ulcer Disease: A Western Australian Perspective

**DOI:** 10.7759/cureus.55194

**Published:** 2024-02-28

**Authors:** Nikitha Boyapati, Vidya Willis, Amanda Foster, David Fletcher

**Affiliations:** 1 General Surgery, Fiona Stanley Hospital, Perth, AUS

**Keywords:** western australia, immunocompromised, non-immunocompromised, antifungals, perforated peptic ulcer

## Abstract

Background

Perforated peptic ulcer disease has a high mortality rate, and there is consensus regarding the use of antifungals in the management of immunocompromised patients; however, there is variability in the utilization of antifungals in the non-immunocompromised cohort. This study aims to describe the current practice related to the use of antifungals in perforated peptic ulcer disease in Western Australia and to determine the peri-operative morbidity and mortality in the immunocompromised and non-immunocompromised cohort receiving antifungals.

Methods

Medical records of patients who underwent surgical repair of perforated peptic ulcer in all Western Australian tertiary hospitals between January 1, 2010, and December 31, 2017, were reviewed retrospectively. Data regarding pre-operative patient factors such as age, gender, and comorbidities, post-operative outcomes such as intra-abdominal sepsis/bleeding, peri-operative antifungal prescription, and abundance of fungal growth on intra-operative samples were collected.

Results

The study included 359 patients. The antifungal prescription was variable. An American Society of Anesthesiologists (ASA) score of 3 or more, presence of pre-operative shock and acidosis, and level of abundance of fungal growth on intra-operative samples were associated with antifungal prescription. Amongst the non-immunocompromised cohort, receiving antifungals was associated with higher morbidity.

Conclusion

The use of antifungals for patients with perforated peptic ulcer disease was variable. An ASA score of 3 or greater and pre-operative shock and acidosis are pre-operative factors predisposing patients to receiving antifungals. There was no difference in morbidity or mortality amongst immunocompromised patients regardless of antifungal prescription or non-prescription. However, in the non-immunocompromised cohort, those who received antifungals had a higher morbidity compared to those who did not.

## Introduction

Peptic ulcer disease (PUD) is a common condition with an incidence of 0.1-0.3% per year and a lifetime prevalence of up to 10% in the general population [1]. The main risk factors for the development of PUD are *Helicobacter pylori* infection, use of non-steroidal anti-inflammatories, cigarette smoking, and gastric bypass surgery [2]. A sharp decline in the incidence of PUD has been observed with the advent of *Helicobacter pylori* treatment; however, the incidence of complications from PUD remains unchanged [2,3].

Perforation and bleeding are the main complications of PUD [4]. Although bleeding is the most common complication [3], perforation has the highest mortality rate of up to 30% [5]. Empiric broad-spectrum antibiotics and operative intervention are the gold standard for managing perforated PUD [6]. The empirical use of antifungals in the management of perforated PUD is a hotly contended topic. Current evidence recommends against the routine use of antifungals due to the lack of morbidity and mortality benefits [6-8].

The World Society of Emergency Surgery (WSES) guidelines recommend that antifungals should be reserved for critically unwell or severely immunocompromised patients and patients with *Candida*-positive peritoneal cultures [6]. A single-centre retrospective study showed that the presence of fungal isolates from peritoneal fluid samples in perforated PUD patients does not impact their peri-operative outcomes [4]. Hence, it can be extrapolated that antifungal therapy may not be necessary in patients with fungal isolates unless they are also critically unwell or severely immunocompromised [4]. However, the outcomes in non-immunocompromised patients treated with antifungals are less known. A systematic review by Huston et al. [9] demonstrated that empiric antifungals are unlikely to improve outcomes in the general population; however, they may improve outcomes in specific patient populations. Apart from the immunocompromised cohort of patients, there may be other patient cohorts who would benefit from antifungals.

Using data collected from all the tertiary hospitals in Western Australia (WA), we aim to describe the current surgical practice related to the use of antifungals in perforated PUD, identify potential pre-operative markers which may improve patient selection for antifungal use, and determine the peri-operative mortality and surgical complications associated with immunocompromised and non-immunocompromised cohort of patients receiving antifungals.

## Materials and methods

All patients aged 18 and above who underwent an emergency surgical repair of perforated peptic ulcer in Fiona Stanley Hospital, Fremantle Hospital, Sir Charles Gairdner Hospital, and Royal Perth Hospital from January 1, 2010, to December 31, 2017, were included. In contrast, patients were excluded if they were under 18 years of age at their index operation, did not present with perforation of a peptic ulcer, had a traumatic or iatrogenic cause of gastric or duodenal perforation, had an isolated bleeding of the peptic ulcer, or did not undergo surgical intervention for the perforation of peptic ulcer.

Following ethics approval from the South Metropolitan Health Service Human Research Ethics Committee and Department of Health Western Australia Human Research Ethics Committee (approval number: RGS0000001195), included patients were selected using relevant International Statistical Classification of Diseases and Related Health Problems, Tenth Revision, Australian Modification (ICD-10-AM) and Australian Classification of Health Interventions (ACHI) 6th to 10th Edition codes to identify those with the correct clinical diagnoses and surgical interventions, respectively. Health information management (HIM) officers at the four hospitals utilized the provided ICD-10-AM and ACHI 6th to 10th Edition codes to retrieve a list of patients. Each of these patients' files were reviewed to ensure they met the abovementioned inclusion criteria.

Data regarding pre-operative factors such as gender, age, American Society of Anesthesiologists (ASA) score, comorbidities, and presence of pre-operative shock and acidosis were collected. Patients were classified as immunocompromised if they had type 2 diabetes, were on immunosuppressant medication or chemotherapy, including transplant recipients, and were suffering from an autoimmune condition or cancer. Data pertaining to peri-operative antifungal prescription, abundance of fungal growth on intra-operative samples, and patient's post-operative outcomes were also collected. Post-operative complications and death were classified according to the Clavien-Dindo (CD) grading system [10,11]. 

Baseline characteristics were expressed as raw numbers, percentages, or an average (range). Logistic regression analysis and Fisher test for independence were used to assess the influence of peri-operative factors on antifungal use and patient outcomes. A p-value of less than 0.05 was considered statistically significant.

## Results

**Figure 1:**
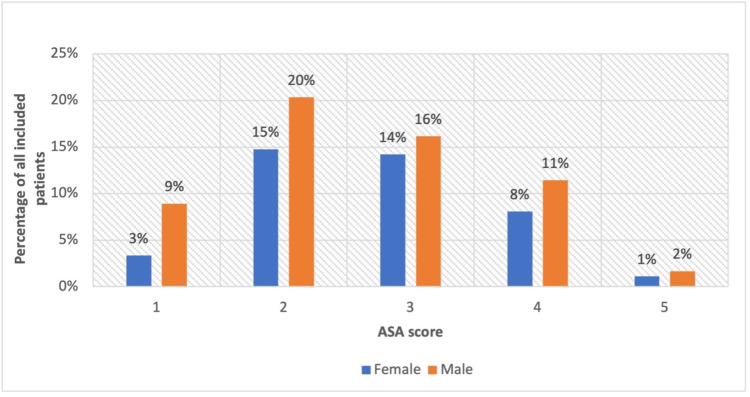
Distribution of ASA scores amongst male and female patients ASA: American Society of Anesthesiologists

**Figure 2:**
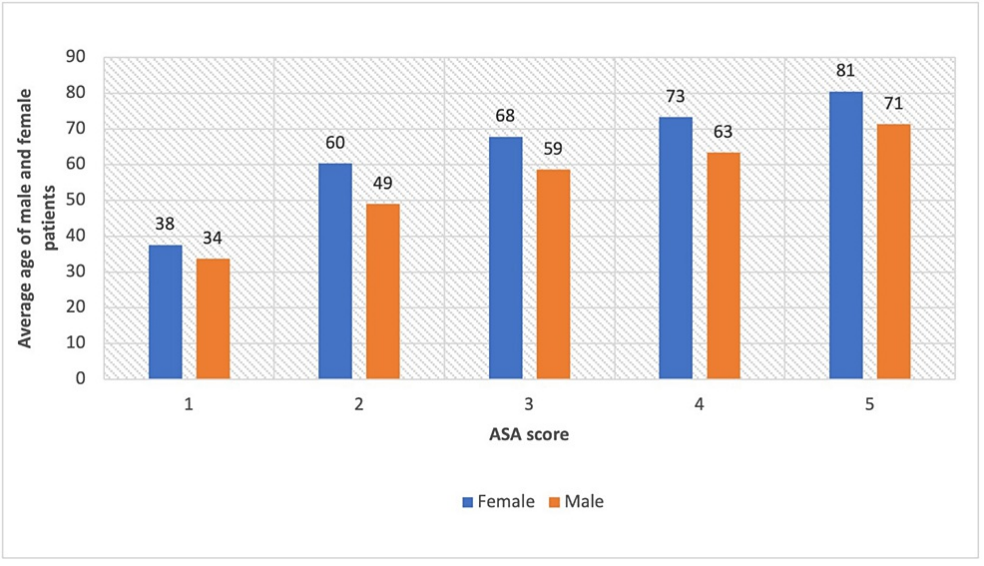
Average age of included male and female patients per ASA score ASA: American Society of Anesthesiologists

Six hundred and twenty-two patients were identified based on the ICD-10 AM and ACHI 6th to 10th Edition codes provided, and 359 patients were included in this study. Two hundred and eight (58%) of the included patients were male. The average age of the male patients was 53 years (range: 18-92), and that of female patients was 64 years (range: 20-98). Two hundred and thirty-three (65%) patients had an ASA score of 2 or 3 (Figure 1). Across all the ASA scores, male patients tended to be younger than female patients (Figure 2).

Two hundred and ninety-two (81%) patients included in the study had a pre-operative venous blood gas performed and only 99 (32%) of these patients had pre-operative acidaemia and 196 (67%) of these patients had an ASA score of 3 or 4. Only 57 (16%) patients included in the study presented with pre-operative shock, and a majority of these patients had an ASA score of 3 or 4.

**Figure 3:**
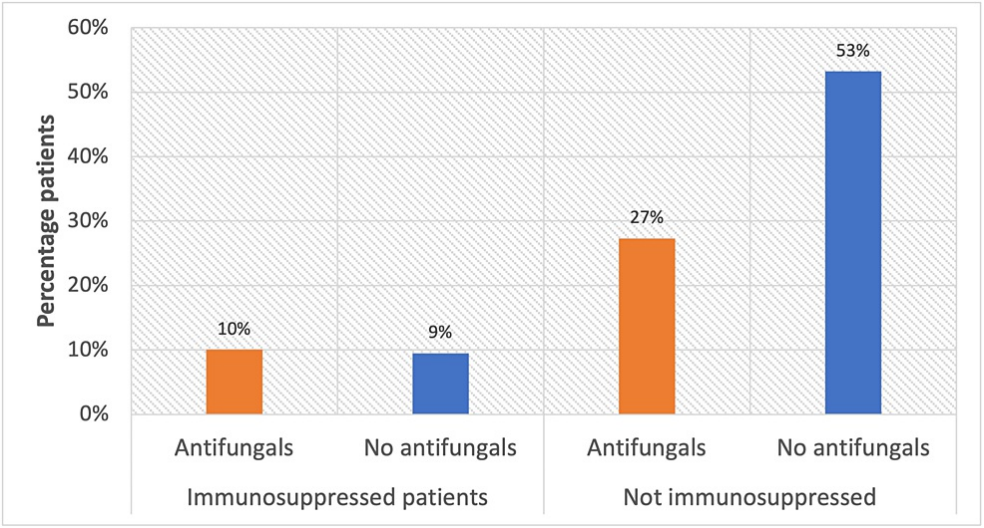
Antifungal prescription depending on immunity status

Antifungal prescription was variable in the study cohort. Due to poor documentation of intent, it was difficult to ascertain which patients received antifungals empirically or were targeted to fungal growth from the intra-abdominal fluid. Only 70 (19%) patients were immunosuppressed in this study, and of these patients, 36 (51%) received antifungals. Of the 289 (81%) patients who were not immunosuppressed, 98 (34%) received antifungals (Figure 3). 

Two hundred and twenty-six (63%) of the included patients had intra-abdominal fluid sent for microscopy, culture, and sensitivity (MCS). Ninety (25%) of the included patients returned a positive fungal culture and received antifungal treatment, and 32 (9%) patients in the study population had a positive fungal culture but were not treated. Forty-three (19%) patients whose intra-abdominal fluid was not sent off for MCS received antifungals, and this was likely empirical. A Fisher test for independence demonstrated that the level of abundance of fungal growth on MCS influenced antifungal prescription (p=0.0392) (Table 1). While a positive fungal culture generally led to antifungal prescriptions, most antifungal prescriptions occurred for those with scanty growth.

**Table 1:** Pattern of antifungal prescription depending on the abundance level on MCS MCS: microscopy, culture, and sensitivity

Abundance level on MCS	Antifungal prescription	
	No (63%)	Yes (37%)
Abundant	3 (0.84%)	13 (3.62%)
Present but not described	6 (1.67%)	11 (3.06%)
Light	6 (1.67%)	10 (2.79%)
Moderate	5 (1.39%)	19 (5.29%)
Scanty	14 (3.90%)	38 (10.84%)
No fungal growth	75 (20.89%)	26 (7.24%)
Not tested	116 (32.32%)	17 (4.74%)

The influence of pre-operative factors like ASA score, gender, Indigenous status, transfer status, and presence of pre-operative acidosis and shock on antifungal prescription patterns was assessed. Only ASA scores and presence of pre-operative shock and acidosis were significantly associated with antifungal prescription (Table 2). Comparison of presence of septic shock and acidosis and ASA scoring as clinical markers of unwellness between the antifungal and non-antifungal prescribed groups found that ASA scores independently were the best predictor of mortality in both the immunocompromised and non-immunocompromised groups. The risk of mortality increased with each increment of the ASA score, more so in the immunocompromised group (Table 3) except for ASA 5.

**Table 2:** Effect of preoperative factors on antifungal prescription practice ASA: American Society of Anesthesiologists

Preoperative factors	Antifungal prescription	P-value
Gender	Independent	0.121
Indigenous status	Independent	1
ASA score	Dependent	0.002
Shock	Dependent	0.008
Acidosis	Dependent	0.0107
Transfer status	Independent	0.509

**Table 3:** Effect of ASA on risk of mortality for immunocompromised and non-immunocompromised patients ASA: American Society of Anesthesiologists; CI: confidence interval

	Immunocompromised patients		Non-immunocompromised patients	
ASA	Predicted probability of death	95% CI for predicted probability	Predicted probability of death	95% CI for predicted probability
1	0.021 (2.1%)	0.002-0.191	0.0007 (0.07%)	0.00007-0.0078
2	0.053 (5.3%)	0.011-0.214	0.005 (0.5%)	0.0011-0.023
3	0.126 (12.6%)	0.056-0.256	0.032 (3.2%)	0.014-0.074
4	0.270 (27%)	0.162-0.415	0.183 (18.3%)	0.1066-0.297
5	0.488 (48.8%)	0.218-0.765	0.596 (59.6%)	0.313-0.828

**Figure 4:**
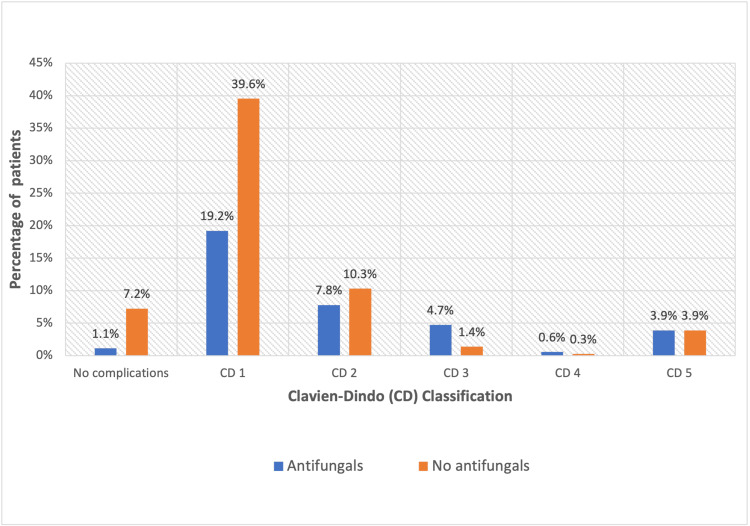
Effect of antifungal prescription on post-operative complications

The antifungal prescription was associated with patients who developed clinical leaks, returned to theatre, and had post-operative ICU admission and a greater risk of post-operative complications (Table 4). Patients receiving antifungals were more likely to sustain grade 1 and 2 complications (Figure 4). Patients who sustained CD 3 complications were more likely to receive antifungals.

**Table 4:** Association between antifungal prescription and post-operative outcomes

Post-operative outcomes	Antifungal prescription	P-value
Intra-abdominal sepsis/bleeding	Dependent	0.0009
Return to theatre	Dependent	0.0002
ICU admission	Dependent	<0.05
Inpatient mortality	Independent	0.159
Number of complications	Dependent	0.0004

There was no statistically significant relationship between CD complication grade and antifungal prescription amongst immunocompromised patients (p-value: 0.98) despite grouping them by ASA scores using the Cochran-Mantel-Haenszel test (p-value: 0.93). Antifungal prescription was also not significantly associated with mortality in the immunocompromised and non-immunocompromised cohort. However, in the non-immunocompromised group, the mean CD grade for the non-antifungal group was 1.19, and the mean for the anti-fungal group was 1.72. This effect size was small but statistically significant with a p-value of 0.00001. 

Non-immunocompromised patients were then stratified according to their ASA scores, and the association between CD grading and antifungal prescription was assessed. ASA score and antifungal prescription were statistically independent of each other, and for a given ASA score, the odds of a patient on antifungals having a higher CD score was 2.23 times (95% CI: 1.34-3.74) than that of a patient not receiving antifungal treatment. The ASA score was the best model to predict the risk of developing complications.

The influence of the abundance level of fungal growth from peritoneal fluid and antifungal prescription on CD grading was assessed after stratifying patients by ASA scores. If the ASA score and abundance level were the same, patients receiving antifungal treatment were 2.27 times more likely to have a complication or death than those not receiving antifungals, but this did not reach statistical significance (95% CI included 1).

## Discussion

Perforated PUD is a significant healthcare issue with a high mortality rate. Hence, it is vital to reach a consensus regarding its management to improve all patient outcomes [6]. This is the first WA study across all three tertiary centres to show that antifungal prescription amongst patients with perforated PUD is variable despite the current literature recommending their use in patients at high risk of fungal infections such as patients with advanced age, with multiple comorbidities, and who are immunocompromised [6,12]. It appears that surgeons generally prescribed antifungals to those they deemed high-risk or unwell albeit with some variation. Seventy (19%) patients were immunocompromised, yet 133 (37%) patients received antifungals and only 36 (51%) immunocompromised patients received antifungal treatment.

Patients who received antifungals typically developed CD grade 3 and 4 post-operative complications comprising clinical leaks, requiring a return to theatre or undergoing radiological intervention, and ICU admission as a result of single- or multi-organ failure. These generally included those who were immunocompromised. Although this practice is in keeping with the WSES and Surgical Infection Society (SIS) guidelines, whether antifungal prescription improved their outcomes is difficult to determine here and requires a prospective assessment which will be fraught with ethical concerns [6,12].

There is conflicting evidence regarding the clinical significance of fungal isolates in peritoneal fluid. Fungi are a vital part of the gut microbiome [13]; however, it is unclear whether the growth of a fungal isolate in a patient with a perforated peptic ulcer indicates contamination versus an infection. Some studies have shown that antifungal therapy is not indicated in healthy individuals with *Candida* in their intra-abdominal fluid as it is cleared with intra-operative abdominal washout without adverse events [14,15]. However, other studies have reported that fungal growth in intra-abdominal fluid is associated with poorer outcomes [16-18].

More recently, studies conducted amongst patients with perforated PUD have shown that the presence of fungal isolates in intra-abdominal fluid does not affect post-operative morbidity and mortality [4,7,9]. No studies have been done to look at the impact of the level abundance of fungal isolate growth on patient outcomes. In this study, antifungals were mostly prescribed for those with scanty growth (Table 1). This together with the low number of prescriptions and retrospective nature of this study makes it difficult to determine if antifungal prescription based on abundance truly improved outcomes. Larger prospective studies are required to determine the impact of intra-abdominal fungal abundance levels and antifungal prescription on patient outcomes. 

While current evidence discourages the use of empiric antifungals [9,19], this study has identified an ASA score of 3 and the presence of pre-operative acidosis and shock as potential markers of critical illness associated with antifungal prescription by WA surgeons. Further studies are required to validate their clinical significance, identify other pre-operative markers which may indicate the use of empirical antifungals, and, most importantly, determine if such practice improves outcomes.

Interestingly, antifungal prescription or non-prescription was not associated with a significant difference in mortality or morbidity amongst the immunocompromised in this study. This finding is consistent with the study conducted by Li et al. [20]. It may be that the comorbidities and poor physiology amongst these high-risk patients were more important factors in determining their outcomes and antifungal prescription did not alter this. On the other hand, it is possible that antifungals prescribed amongst the worse of the high-risk patients confounded the level of benefit within the immunocompromised group compared to those not receiving antifungals. Moreover, the patient population in this study may be too small to demonstrate the effect of antifungals on outcomes.

Antifungal prescription amongst the non-immunocompromised group, however, was associated with higher morbidity even after patients were stratified by ASA scores albeit a small difference. This is likely due to clinicians already recognising unwell patients and prescribing antifungals rather than antifungal prescriptions causing increased morbidity. Antifungal prescription here is therefore a proxy for clinical unwellness, and the small but significant difference seen in outcomes is likely due to antifungals reducing the severity of CD complications experienced by these patients.

This study has therefore highlighted that antifungal prescription in such non-immunocompromised patients may alter outcomes and further studies are required to identify physiological markers to indicate who should receive them and their effectiveness in improving patient outcomes. This study has highlighted abundance levels in peritoneal fluid, ASA scores 3 and above, pre-operative shock, and acidosis as such markers that should be further investigated. Despite identifying additional clinical factors that may require antifungal prescription, it is important to avoid the indiscriminate use of antifungals due to emerging antifungal resistance isolates [21]. This study has also shown that the ASA score is likely to be the best model of morbidity and mortality prediction which is already well established in current practice.

The limitations of this study include its retrospective nature and associated poor documentation leading to missing data and difficulty in determining the extent of empirical antifungal prescription. There was also variability in the practice of testing for acidosis or performance of peritoneal fluid microscopy, potentially missing patients who presented with acidosis or had intra-abdominal fungal growth in this study. The number of patients prescribed antifungals was also small.

## Conclusions

The use of antifungals for patients with perforated PUD varies amongst general surgeons in WA. ASA score of 3 and above and the presence of pre-operative acidosis and shock should raise awareness for the potential severe critical illness post-operatively which may warrant antifungal prescription, particularly in the non-immunocompromised group. Further studies assessing patient outcomes following antifungal prescription based on clinical status and abundance of intra-abdominal fungal growth as opposed to immune status alone would aid future decision-making in the post-operative period. It is agreed that the indiscriminate use of antifungals is unlikely to improve patient morbidity or mortality but in fact contributes to emerging resistance.
